# TRP matters: a pilot study on expression of mRNA in the parotid glands and their benign tumors

**DOI:** 10.1016/j.bbrep.2026.102696

**Published:** 2026-07-07

**Authors:** Lukas Louis, Gentiana Wenzel, Alessandro Bozzato, Thomas Tschernig, Stefan Wagenpfeil, Bernhard Schick, Silke Wemmert

**Affiliations:** a Institute of Anatomy and Cell Biology, Saarland University, Kirrberger Straße, Homburg/Saar, 66421, Germany; b Department of Otorhinolaryngology, Saarland University, Homburg/Saar, Germany; c Institute for Medical Biometry, Epidemiology and Medical Informatics, Saarland University, Saarland, Saarbrücken, Germany

**Keywords:** TRP channels, Salivary glands, Warthin tumor, Pleomorphic adenoma

## Abstract

**Purpose:**

The transient receptor potential (TRP) channels are a group of nonselective cation channels, which play critical roles in a variety of physiological processes and are also involved in the development of various carcinomas. However, the presence of TRP channels and their role in human benign salivary gland tumors has not been investigated yet.

**Methods:**

We assessed the mRNA expression of the TRP channels TRPA1, TRPC3, TRPC6, TRPM4, TRPM8 as well as the channel associated factors TCAF1 and TCAF2 in human specimens of pleomorphic adenoma (PA, n = 10) and Warthin tumors (WT, n = 10) of the parotid gland. The control group consisted of 5 healthy human parotid gland (PG) specimens. The analysis has been performed using qRT-PCR.

**Results:**

The mRNAs of TRPM4, TRPC6, TCAF1 and TCAF2 were found in normal parotid gland tissue and even more pronounced in PA and WT. However, no mRNA of TRPA1, TRPC3 and TRPM8 could be detected in normal parotid tissue nor in WT. In contrast, TPRC3 and TRPM8 mRNA were detected in PA. In 4 out of 10 PA high mRNA expression levels were found for these two genes.

**Conclusion:**

Our current pilot study suggests that TRPM4, TRPC6, TCAF1 and TCAF2 are expressed in normal parotid gland tissue and partially elevated in PA as well as WT of the parotid gland. TRPC3 and TRPM8 seem to be restricted to PA and may be highly expressed in a subgroup of parotid gland PA. These findings are important for a better understanding of PA biology.

## Highlights

• First report on distinct TRP-channel and associated factor genes in parotid glands/benign tumors.
• TRPM4, TRPC6, TCAF1 and TCAF2 gene expression more pronounced in benign tumors.
• TRPC3 and TRPM8 mRNA found exclusively in a subgroup of pleomorphic adenoma.
• Pilot study which opens the door for further research and therapies at the horizon.

## 1 Introduction

Salivary gland neoplasms are a rare and heterogeneous group among head and neck tumors. More than 80% of surgically treated tumors are benign, depending on the gland treated. Here, pleomorphic adenoma (PA) and Warthin's tumor (WT) are the most common benign salivary gland tumors, which together account for more than 90% of all benign parotid tumors [1]. While the pathogenesis of PA is mostly unknown, numerous etiological factors have been suggested in WT: chronic inflammation, Epstein Barr virus infection, radiation, genetic changes and most importantly cigarette smoking [2].

Both most common benign parotid gland tumors clearly differ clinically. PAs may recur, can undergo malignant transformation and are associated with worse overall survival [3], whereas malignant transformation or recurrence of WT seldom occurs. Therapy of recurrent PA may pose a surgical challenge especially in multilocular presentation. In these cases, even tumors that underwent radiotherapy may show progression later, especially when radiotherapy is performed in younger ages [4]. New treatment options of recurrent PA are therefore required.

Transient receptor potential (TRP) channels are a family of non-selective cation channels which have been subject of scientific research following their first description in retina cells of the drosophila fly in 1978 [5,6]. Since then, there have been multiple studies linking different subtypes of TRP channels to tumor growth in various carcinomas such as mamma carcinoma [7], pancreatic carcinoma [8], adenocarcinoma of the lung [9], prostate cancer [10] and many more. Some of these studies also discuss a potential prognostic correlation between an upregulated TRP expression and a higher patient mortality rate. While most TRP channels are non-selective, their permeability for calcium cations in particular has been associated with their role in tumor genesis [11] and tumor vascularization [12]. There have also been studies discussing the potential targeting of TRP channels in anti-cancer drugs [13].

To the best of our knowledge, no studies addressing TRP channels in benign human salivary gland tumors have been performed so far. Therefore, we aimed to identify the mRNA expression of a panel of TRP channels TRPA1, TRPC3, TRPC6, TRPM4, TRPM8 as well as the TRPM8 channel associated factors TCAF1 and TCAF2 in human specimens of PA and WT. The control group consisted of healthy human parotid gland specimens. The analysis has been performed by qRT-PCR and the results were correlated to the clinical parameters.

## 2 Methods

### 2.1 Tissue samples

This retrospective study consisted of 20 patients with benign salivary gland tumors (mean age 50.2, range 14 - 76). All patients underwent parotid tumor surgery at the Department of otorhinolaryngology at the Saarland University Medical Center (Homburg, Germany). Tumors were histologically diagnosed as pleomorphic adenomas (n = 10, mean age 45.2, 6 male/4 female) and Warthin tumors (n = 10, mean age 55.2, 7 male/3 female). As a control group, normal parotid specimens from 5 patients (mean age 58, 2 male/3 female) were available. The tissue was snap frozen immediately after surgery and stored in liquid nitrogen until further dissection for this study. The use of human tissues has been performed according to the Code of Ethics of the World Medical Association (Declaration of Helsinki) as well as approved by the Institutional Review Board (index number 218/10) at the Saarland University.

### 2.2 Quantitative real-time PCR

Tumor tissue was homogenized, and total RNA was extracted using the RNeasy Mini Kit (Qiagen, Hilden, Germany) according to the manufacturer's guidelines. RNA concentration and purity were assessed with the Nanodrop spectrophotometer (Thermo Scientific, Massachusetts, USA) and 500 ng RNA was reverse transcribed to cDNA using Superscript IV Vilo kit (Thermo Fisher Scientific, Massachusetts, USA). RT-qPCR was performed in technical triplicates using specific TaqMan Gene Expression Assays (Thermo Fisher Scientific, Massachusetts, USA) on an ABI StepOne Plus™ instrument (Thermo Fisher Scientific, Massachusetts, USA). The primers employed in our study are listed in Table 1. The thermal profile for qPCR was 20 s pre-incubation at 95°C for one cycle, followed by 40 cycles of 95°C for 1 s and 60°C for 20 s. For relative quantification, fold change for targeted genes was normalized to the housekeeping genes β2M and GAPDH according to 2^−ΔΔCT^ method.

**Table 1:** Taqman Gene Expression Assays (for details see: https://www.thermofisher.com/de/de/home/life-science/pcr/real-time-pcr/real-time-pcr-assays.html with links to the National Library of Medicine, GenBank, Nucleotide).

Taqman Primer	Assay-ID
**TRPC3**	Hs00162985_m1
**TRPC6**	Hs00988479_m1
**TRPA1**	Hs00175798_m1
**TRPM4**	Hs00214167_m1
**TRPM8**	Hs01066596_m1
**TCAF1**	Hs00206993_m1
**TCAF2**	Hs04186295_m1
**B2M**	Hs00187842_m1
**GAPDH**	Hs02758991_g1

### 2.3 Data analysis

GraphPad Prism version 9 (GraphPad Software, Boston, USA) was used for data display and statistical analysis. Formal inferential statistical analyses of comparisons between PA and parotid gland, WT and parotid gland, age groups, and patient sex were not performed due to sample sizes. However, we present descriptive statistics as well as respective scatter plots.

## 3 Results

**Fig. 1:**
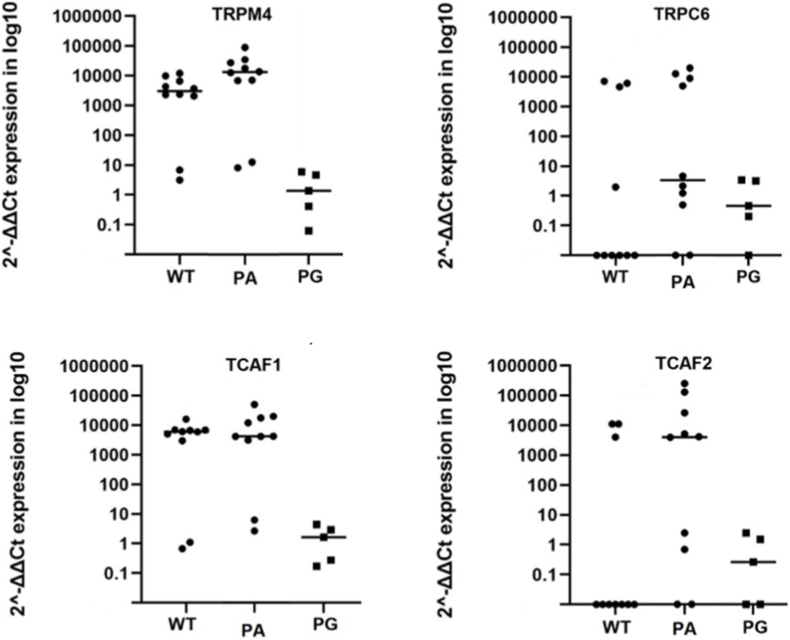
**Relative mRNA-expression of different TRP channel genes and factors in Warthin tumors (WT) and pleomorphic adenomas (PA) compared to normal parotid gland (PG) tissues.** Real-time PCR analysis of TRPM4, TRPC6, TRPM8 Channel Associated Factor 1 (TCAF1) and TRPM8 Channel Associated Factor 2 (TCAF2) in WT (n = 10) and PA (n = 10) compared to normal PG (n = 5). Relative expression of all target genes was calculated according the 2^−ΔΔCt^ method and β2-Microglobulin and GAPDH were used as endogenous genes.

To identify the role of TRP channels and TRPM8 associated factors in WT and PA, we analyzed the mRNA expression of TRPA1, TRPC3, TRPM4, TRPC6, TRPM8, TCAF1 and TCAF2. In the normal parotid gland, no expression of TPRA1, TPRC3 and TRPM8 was observed. In contrast, the genes of TRPM4, TRPC6, TCAF 1 and TCAF2 were found in in normal parotid glands and even more pronounced in WT and PA (Fig. 1). In general, a heterogeneous expression in-between the specimens was observed ranging from no detectable mRNA expression up to a detectable expression of the respective genes (Fig. 1).

No differences could be noticed comparing the expression of TRPM4, TRPC6, TCAF1 and TCAF2 between WT and PA. Also, no correlation to clinical parameters like patient sex or age was observed. The age threshold for the comparative groups was set in accordance to the mean age of our PA (45 y) and WT (55 y) cohorts.

### 3.1 mRNA expression of TRPC3 and TRPM8 in PA

**Fig. 2:**
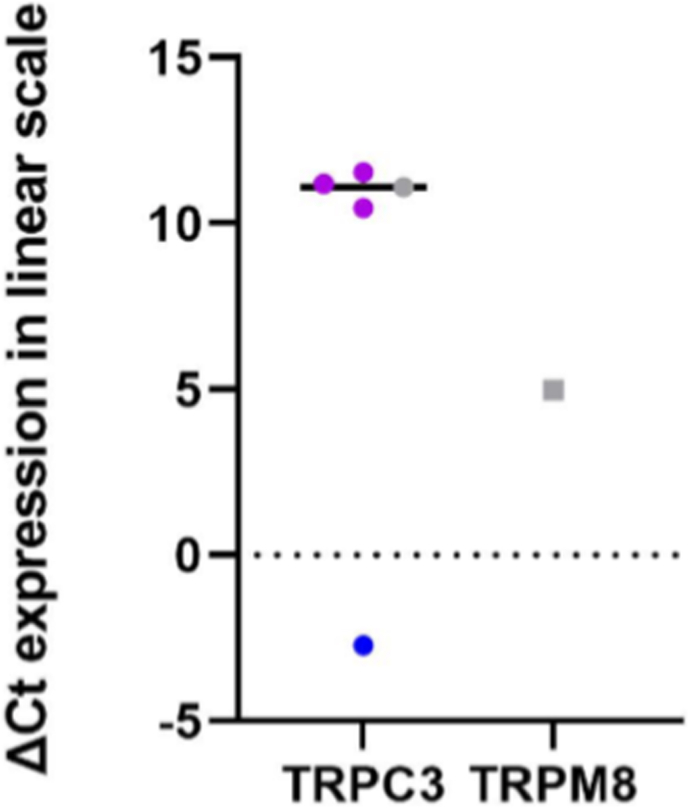
**Relative expression level of TRPC3 and TRPM8 in PA**. mRNA expression of the TRP channels. Results are shown in a linear scale; hereby a higher ΔCt represents a lower mRNA expression. Grey markings indicate the mRNA expression of the same PA patient.

In 5 PA, mRNA expression of TRPC3 was observed, one of which presented a very strong upregulation of this channel. Notably, one of the samples with TRPC3 mRNA also showed an TRPM8 mRNA (grey markings, Fig. 2).

## 4 Discussion

While the distribution of TRP channels in the human body has been investigated in various studies [14], their expression in pleomorphic adenoma (PA) and Warthin's tumor (WT) has not been examined so far. This is the first study showing TRP channel mRNA expression in two benign parotid tumors of the human body. TRP channels have often been suspected of taking part in calcium pathways leading to stronger cell proliferation [9,[15], [16], [17]] and thus being involved in the tumorigenesis of different organs [[7], [8], [9], [10]]. The mRNA expression of the different TRP-channels varies in different tumor entities, which makes it difficult to attribute a specific function to them in tumorigenesis.

TRPM4 and TRPC6 have already been associated with tumor growth in several malignant tissues, such as prostate cancer, liver cancer, breast cancer and many more [13,18,19]. Moreover, in colorectal cancer [20] TRPM4 (over)expression was shown to be associated with more aggressive tumor features, whereas the knockout of TRPM4 has led to reduction of invasiveness and migration of the colorectal cancer cells. This observation determined scientists to perform further studies that unrevealed promising candidates for the treatment of colorectal cancer by targeting TRPM4 [13,21]. Our data suggests a detectable expression of the TRPM4 gene in WT as well as in PA. A role of TRPM4 in growth rate and prognosis might be speculated. Another speculation might be that higher expression rates indicates later degeneration which has been reported for both tumors [3,[22], [23], [24]]. There, it is a weakness of this study that the TRP proteins were not investigated.

The TRPC6 protein was already demonstrated in the submandibular and lacrimal gland as well as in malignant salivary gland tumors by immunohistochemistry [25]. In accordance with our results, not all investigated samples showed positive staining in the cited study. This is most likely due to tumor heterogeneity occurring between and in the individual samples. Our preliminary data demonstrated a detectable expression of TRPM4 and TRPC6 mRNA in benign salivary gland tumors. Therapeutics derived from the mentioned candidates [13,21] might therefore be effective against WT and PA. However, as TRPM4 and TRPC6 show a broad expression range in both tumor and healthy tissues, a thorough investigation of the potential side effects of TRP inhibitors on healthy tissues and organs is therefore of great importance [13,26,27]. TRPC3 is already known to promote growth in ovarian carcinoma [28] and its impact has also been shown in triple negative breast cancer cells, where a blockade of TRPC3 lead to induction of apoptosis and sensitization to chemotherapy [29,30]. We found mRNA expression of TRPC3 in some, but not all, PAs, which was not the case in WT and healthy parotid gland tissues. As the channel is not expressed in WT or healthy parotid tissue, this observation might give impact for diagnostical purposes. As PA can undergo malignant transformation, further studies with carcinoma ex pleomorphic adenoma (CXPA) should address a potential role for TRPC3 in this context.

TRPM8 Channel Associated Factor 1/2 (TCAF1/TCAF2) proteins antagonistically regulate the cold-sensor protein TRPM8. In multiple carcinomas, including lung cancer, gastric cancer and skin cancer [31], an upregulation of TRPM8 has been related to higher proliferation and metastasis of tumor cells [8,31]. In prostate cancer, the role of TRPM8 channels seems controversial due to reports showing both pro-proliferative/antiproliferative and pro-apoptotic/anti-apoptotic effects of the channel, proposed to be related to differences in androgen-sensitivity of the cells [31].

In our cohort of human samples, we identified an expression of TCAF1 and TCAF2 in WT and PA as compared to healthy parotid gland whereas TRPM8 expression was only present in one PA. Therefore, a similar correlation as in the mentioned cancers seems not likely in the majority of our cases, leading to the assumption that TCAF1 and TCAF2 might interact with other cellular structures or TRP channels to facilitate proliferation. However, while the overall risk of malignancy is relatively low, distinct molecular sub-groups seem to have an increased risk of malignant progression to carcinoma ex pleomorphic adenoma [32]. Considering the higher degenerative rate of PA, it could be interesting to investigate whether a TRPM8 and/or TRPC3 expression leads to higher rates of tumor degeneration. Therefore, further studies must elucidate the interactions of TRP and TCAF1/2 expression to promote or decrease proliferation, especially in cancers with malignant transformation derived from PA and WT, such as CXPA [3] and malignant lymphoma [22]. While the latter has only been reported in a few cases of degenerated WT, CXPA can degenerate from untreated PA with a mortality rate of 55%. Due to the risk of metastasis [24], systematic treatment could be advantageous over localized therapy such as surgery and radiation.

The role of TRP channels in physiological and pathological tissue has been a much-debated topic in recent research. Since our data suggests an expression of TRPC6, TRPM4, TCAF1 and TCAF2 in certain samples of PA and WT these channels should be investigated further. However, there is a pronounced heterogeneity ranging from expression to no-expression of the investigated TRP channels in individual entities. Most likely, the expression differences of certain targets might be associated with higher degenerative or higher proliferative rates or reflect the intra-heterogeneity of the analyzed sample. One could speculate that future therapeutics target these channels. Alternatives are preferable because parotidectomy has possible harmful side effects like facial paralysis or Frey syndrome [33]. In a clinical context, testing the individual tumors prior to therapy would be a step to personalized medicine.

## 5 Conclusion

The presented pilot is a study focusing only on mRNA expression, with small sample sizes but found the mRNA for the investigated TRP channels and TCAF1/2 in WT and PA. Despite the methodological limitations and the small number of cases, these findings might inspire further research on salivary gland neoplasms.

## Author's contributions

BS, AB and TT conceived and designed the study. LL, GW and SiWe performed the study and collected the data. StWa contributed the statistical analysis. LL and SiWe wrote the paper and all authors critically revised it.

## Declaration of competing interest

The authors declare that they have no competing interests.

## Data Availability

Data will be made available on request.
